# The National Health Bill 2009 and afterwards

**DOI:** 10.4103/0972-2327.53074

**Published:** 2009

**Authors:** Sanjeev V. Thomas

**Affiliations:** Professor of Neurology and Editor, Annals of Indian Academy of Neurology, Department of Neurology, SCTIMST, Trivandrum, India. E-mail: editor@annalsofian.org

The Government of India took a landmark decision when it decided to introduce the National Health Bill, 2009.[1] The bill recognizes health as a fundamental human right and states that every citizen has a right to the highest attainable standard of health and well-being. The constitution of India, under Articles 14, 15, and 21, recognizes the right to life as a fundamental right and places obligations on the Government to ensure protection and fulfillment of the right to health for all, without any inequality or discrimination. The basic tenets of the Bill include the peoples' right to health and healthcare, the obligations of the governments and private institutions, core principles/norms/standards on rights and obligations, the institutional structure for implementation and monitoring, and the judicial machinery for ensuring health rights for all. The bill provides itemized lists of the obligations of the central and state governments. Chapter III of this bill provides elaborate rights to health care, including terminal care, for everyone. A heartening point is that the bill guarantees that no person shall be denied care under any circumstances, including the inability to pay the requisite fee or charges. Prompt and necessary emergency medical treatment and critical care must be given by the concerned health care provider, including private providers. As per the bill, the health care provider, including the clinician, would be obligated to provide all information to the patients regarding the proposed treatment (risks, benefits, costs, etc.) and any alternate treatments that may be available for the particular condition/disease. There is a clause in this chapter that demands that the user (i.e., the patient) respect the rights of the health care providers by treating them with respect, courtesy, and dignity and refrain from any abuse or violent or abusive behavior towards them or to the rights provided to them. The bill envisages the establishment of National- and State-level Public Health Boards to formulate national policies on health, review strategies, and ensure minimum standards for food, water, sanitation, and housing. These boards would also lay down minimum standards and draw up protocols, norms, and guidelines for diverse aspects of health care and treatment. The bill provides for elaborate mechanisms for monitoring at the government and community levels. There is a need to have wider discussion on the scope and activities of these monitoring agencies and regarding dispute resolution and redressal mechanisms listed in chapter V of the Bill.

A comprehensive Act that covers the various aspects of health care rights, delivery and related matters has been a pressing need in this country for long. Several international and national agencies, as well as the Honorable Supreme Court of India, have drawn the attention of the Government to this issue. The Bill, once enacted, would have far-reaching consequences on the Indian health scenario. It would demand greater levels of professionalism, standards of care, accountability, from the care providers and, besides, ensure protection for them. The Bill calls for greater official involvement of professional bodies like the Indian Academy of Neurology in the health care management in this country. It is hoped that professional bodies will carefully study this Bill and come out with suggestions and recommendations as soon as possible.

I also take this opportunity to draw the attention of the readers and the members of the academy to the special announcement regarding the retraction of one of the papers published in our journal. Progress in medical science, like in any other scientific discipline, is founded on precise and honest observations and studies. Medical journals provide a platform for scientists to bring their original work before the rest of the world. Unfortunately, there are occasions when persons or groups attempt to violate the basic tenets of publication ethics by resorting to unscrupulous and unethical means. We consider this a serious offence and denounce any such efforts. We also wish to emphasize that we will continue to be vigilant toward such malpractices and will strive to bring the culprits to justice if any such attempts are detected. Our journal is now indexed with several databases and is accessible in full text all across the world. Readers and fellow scientists who detect plagiarism or other such offences should bring it to our notice. Let us make every endeavor to promote science in its true spirit.
